# Credibility assessment of cold adaptive *Pseudomonas jesenni* MP1 and *P. palleroniana* N26 on growth, rhizosphere dynamics, nutrient status, and yield of the kidney bean cultivated in Indian Central Himalaya

**DOI:** 10.3389/fpls.2023.1042053

**Published:** 2023-01-30

**Authors:** Amir Khan, Ajay Veer Singh, Navneet Pareek, Pratima Arya, Viabhav Kumar Upadhayay, Arun Kumar Jugran, Pankaj Kumar Mishra, Reeta Goel

**Affiliations:** ^1^ Biofortification lab, Department of Microbiology, College of Basic Sciences and Humanities, Govind Ballabh Pant University of Agriculture and Technology, Pantnagar, India; ^2^ Department of Soil Science, College of Agriculture, Govind Ballabh Pant University of Agriculture and Technology, Pantnagar, India; ^3^ Department of Microbiology, College of Basic Sciences and Humanities, Dr. Rajendra Prasad Central Agriculture University, Samastipur, India; ^4^ G. B. Pant National Institute of Himalayan Environment (GBPNIHE), Garhwal Regional Centre, Srinagar, India; ^5^ ICAR-Vivekananda Parvatiya Krishi Anusandhan Sansthan, Almora, India; ^6^ Institute of Applied Sciences and Humanities, GLA University, Mathura, India

**Keywords:** Biofortification, cold-adaptive PGPR, hill agriculture, kidney bean, microbial diversity, nutrients

## Abstract

Kidney bean (*Phaseolus vulgaris*) productivity and nutritional quality are declining due to less nutrient accessibility, poor soil health, and indigent agronomic practices in hilly regions, which collectively led to a fall in farmer’s income, and to malnutrition in consumers. Addressing such issues, the present investigation was designed to assess the impact of *Pseudomonas jesenii* MP1 and *Pseudomonas palleroniana* N26 treatment on soil health, microbial shift, yield, and nutrient status of the kidney bean in the Harsil and Chakrata locations of Indian Central Himalaya. *P. jesenii* MP1 and *P. palleroniana* N26 were characterized as cold adaptive PGPR as they possessed remarkable *in vitro* plant growth promoting traits. Further, field trial study with PGPR treatments demonstrated remarkable and prolific influence of both strains on yield, kidney bean nutrient status, and soil health at both geographical locations, which was indicated with improved grain yield (11.61%–23.78%), protein (6.13%–24.46%), and zinc content (21.86%–61.17%) over control. The metagenomic study revealed that use of bioinoculants also concentrated the nutrient mobilizing and plant beneficial microorganisms in the rhizosphere of the kidney bean. Moreover, correlation analysis also confirmed that the plant growth-promoting traits of *P. jesenii* MP1 and *P. palleroniana* N26 are the basis for improved yield and nutrient status of the kidney bean. Further, cluster and principal component analysis revealed that both P*. jesenii* MP1 and *P. palleroniana* N26 exhibited pronounced influence on yield attributes of the kidney bean at both the locations. At the Harsil location, the *P. jesenii* MP1-treated seed demonstrated highest grain yield over other treatments, whereas at Chakarata, *P. jesenii* MP1, and *P. palleroniana* N26 treatment showed almost equal enhancement (~23%) in grain yield over control. The above results revealed that these bioinoculants are efficient plant growth promoters and nutrient mobilizers; they could be used as green technology to improve human health and farmer’s income by enhancing soil health, yield, and nutrient status of the kidney bean at hilly regions.

## 1 Introduction

Planet earth is considered a cold planet as the major geographical region encounters the average temperature of 15°C across the year (Dhakar and Pandey, 2020). Likewise, the Himalayan region is vastly distributed (>2000 km plain region and >7000 msl height) among many countries, which demonstrates unique properties such as gradient of temperature over altitudinal variation and exclusive and exceptional massive microbial diversity. The Himalayan region demonstrates a diverse environment over different altitudes and hence their soil quality and native growing crops are also diverse. The Indian Himalayan region is known as the reservoir of microbial diversity, but the hilly area’s soil is getting shattered through various natural and anthropogenic activities, which ultimately reduce the soil health, crop productivity, diversity sustenance, nutrient availability, and increased disease occurrence and health hazards (Babu et al., 2020; Kc et al., 2022). Among the numerous crops cultivated in the Himalayan region, the kidney bean (*Phaseolus vulgaris* L.) crop is known for its nutritional richness and premium quality (Jan et al., 2021). It serves as a source of food security and nutrition for low-income people in rural and urban regions. Protein, complex carbohydrates, vitamin B elements (thiamin, folic acid, and niacin), and micronutrients (iron, zinc) are all abundantly present in kidney beans. In India the average yield ranges from 421–1,000 kg/h, which is relatively lower compared to the international average yield ranging from 1,500 to 2,000 kg ha^-1^ (Gupta et al., 2019; Xu et al., 2022). The soil health of hills is steadily deteriorating due to the unavailability of macro and micronutrients, which anonymously reduced kidney bean yield, nutrient content, and quality. Hence, the reduced production and low nutrient value crops are the basic reason for the poor economy of farmers, hunger, and malnutrition (Khan et al., 2020a). As the population is steadily increasing and crop production is constant, it will emerge as a huge problem in the future. Addressing such issues, one of the efficient and sustainable approaches is the implication of potential cold adaptive plant probiotic bioinoculant application in the Himalayan field that can confer the nutrient availability and maintain the soil health status in a sustainable manner. Below 20°C mesophilic bacterial metabolism and transportation get restricted, which results in the reduced functioning, whereas cold adaptive bacteria possess various adaptation strategies to overcome such problems (Nedwell, 1999; Tribelli and López, 2018). Therefore, in order to work efficiently in hilly regions where temperature is low, the bacteria should be adaptive to cold environments.

The microorganisms are the prevalent and diverse living entities inside the soil and are fundamentally responsible for organic matter decomposition and soil nutrient recycling and are vital for soil health and ecosystem functioning. Microbes isolated from the hilly regions generally exhibited plant growth-promoting traits with the cold adaptive property (Puranik et al., 2022). Furthermore, these are supposed to be the best probiotics for improving plant growth performance, production, and nutrient mobilization in the cold environment of the hill region. In the present study, the potential bioinoculants, i.e. *P*. *jesenii* MP1 and *P*. *palleroniana* N26 (**Table S1**), were cold adaptive diazotrophic phosphate solubilizers and initially isolated from the hilly regions of Munsyari and Nainital, respectively (Tomer et al., 2017). Moreover, these bacterial strains demonstrated their remarkable impact on soil health and plant production improvement (Tomer et al., 2017; Rajwar et al., 2018; Joshi et al., 2019). Based on the previous findings, *P. jesenii* MP1 and *P. palleroniana* N26 were applied as bioinoculants with the kidney bean to assess their impact under field conditions at two diverse geological locations (i.e. Harsil and Chakrata) of Indian Central Himalaya. *P. jesenii* MP1 and *P. palleroniana* N26 were introduced in the kidney bean field through seed treatment and investigated for nutrient recycling and acclimatization, yield enhancement potential, and their effect on the microbiome and functional gene profile shift in two different geographical locations of Indian Central Himalaya.

## 2 Material and methods

### 2.1 Bacterial strains and growth conditions

Two bacterial strains i.e., *Pseudomonas jesenii* MP1 and *Pseudomonas palleroniana* N26, native to hill regions were obtained from the departmental culture collection of G. B. Pant University of Agriculture and Technology, Pantnagar, Dist. Udham Singh Nagar, Uttarakhand (**Table S1**). Both strains can grow on nutrient agar medium at 28°C and are already characterized for phosphate solubilization and nitrogen transformation potential (Tomer et al., 2017; Rajwar et al., 2018; Joshi et al., 2019). The strains were retrieved and preserved in slants and glycerol stock for further use at 4°C and -20°C, respectively.

### 2.2 Estimation of PGP properties of bacterial strains

To assess the plant growth-promoting (PGP) ability of bacterial strains, multiple PGP properties have been estimated under *in vitro* conditions. The zinc solubilization potential of test bacterial strains was determined in a basal liquid medium supplemented with a zinc source (ZnO as the insoluble Zn source) through atomic absorbance spectroscopy (Upadhayay et al., 2022a). The Siderophore production efficiency of bacterial strains was estimated using the CAS dye method described by Schwyn and Neilands (1987). Moreover, the potassium mobilization potential of the strains was measured with flame photometry (Rajawat et al., 2014). Further, exo-polysaccharide production was also examined by the protocol described by Siddikee et al. (2011). The biofilm production ability of test bacterial cultures was examined through the ring formation method and further estimated by dissolving ring in glacial acetic acid and absorbance was measured at 570 nm (Lotfi et al., 2014), While bacterial IAA production was estimated by the method described by Gordon and Weber (1951), followed by Khan and Singh (2021) who have used (100 µg/ml) tryptophan concentration as a precursor. A qualitative test for ammonia production was performed as described by Cappuccino and Sherman (1992).

### 2.3 Field and experiment locations

To assess the impact of bacterial strains on kidney bean production, nutrient assimilation, and soil health improvement, a field study was performed during May to September, 2020 in the farmer’s fields positioned in the Indian Central Himalayan region of the two geographically different locations of Chakrata (30.7016˚N, 77.8696˚E; 2100 msl) and Harsil (31.0383˚N, 78.7377˚E; 2700 msl). Both the locations hold moist temperate climatic zone with an average temperature of 8°C–29°C during May to September and average annual rainfall of 950–1300 mm, due to which these are the highly recommended locations for kidney bean cultivation in the hill regions.

### 2.4 Seeds treatment and sowing

The present experiment was conducted with two potential cold adaptive di-azotrophic phosphate solubilizing bacteria, i.e., *P. jesenii* MP1 and *P. palleroniana* N26. The experiment was conducted at farmer fields, where the agriculture land is in fragmented and terraced form (the average field dimension area was equal or higher than 150 m^2^). The bacterial inoculated fields served as treatment and uninoculated farmers’ practices as control. The bioformulation of potential bioinoculants (1 × 10^8^ CFU/g) was prepared using charcoal as carrier material by mass multiplication of bioinoculants for 24 h. Kidney bean seeds were bacterized with charcoal-based bioformulation (1 × 10^8^ CFU/g) at the rate of 10 g/kg seeds (Germination percentage 90%). Afterward, the seeds were dried under the shade to adhere with bioinoculants, and seeds were sown immediately in the 18 fields at the Harsil and Chakrata locations. Simultaneously, uninoculated control fields were also maintained at both locations.

### 2.5 Soil, plant, seed sampling, and analysis

Field soil was having sandy clay loam texture with a pH of around 6.8 to 7.5 at both locations. Harsil soil was rich in organic carbon compared to Chakrata soil. Before sowing and at harvest, soil samples were collected in sterile poly bags by digging the field through a trowel for a depth of 0–20 cm at five random locations from each field, then taken to the laboratory and stored at -20°C for further analysis. To assess the impact of bioinoculants, plants, and grain and yield data were collected from each treated and control field at the harvesting stage. All the agronomical attributes and yield parameters were determined and recorded as per the standard procedures.

### 2.6 Seeds nutrient analysis

#### 2.6.1 Estimation of zinc and iron in seed samples

After harvesting, collected seeds were analyzed for available Zn and Fe content. For this, grains were subjected to digestion on hotplate with a tri-acid mixture [Nitric acid (10): Perchloric acid (4): Sulfuric acid (1) v/v/v] following the addition of 5 ml 6 N HCl in the digestion mixture. Then the flask volume was made up to 50 ml by adding deionized water. Subsequently, the mixture was filtered and passed through filter paper (Whatman no. 42). Afterward, digested and filtered material was inspected for available Zn and Fe content with the help of an atomic absorption spectrophotometer (Estefan et al., 2013).

#### 2.6.2 Protein, carbohydrate, methionine and antioxidant activity estimation in seed samples

Protein content in the seed sample was estimated through the Bradford method. In brief, the whole protein was extracted from the seed sample. The extent of protein content in the sample was measured based on its ability to form a complex with Coomassie brilliant blue G-250 dye. Further, the protein content was estimated by extrapolating the optical density of samples on a known concentration standard curve of protein (Sadasivam, 1996). Moreover, carbohydrate content was determined through the Anthrone method. In brief, seed carbohydrate was hydrolyzed into monosaccharide by acid hydrolysis. Further, the Anthrone reagent was added to form a green color reagent monosaccharide complex following the absorbance recorded at 630 nm. Afterward, carbohydrate content was determined by extrapolating the optical density on a known concentration standard curve (Sadasivam, 1996). The seed’s antioxidant activity was estimated through extract preparation of the seed samples by adding 80% methanol. Further, the extract was centrifuged and added with DPPH following incubation for 30 min in the dark. Finally, the samples were read at 517 nm and antioxidant activity was calculated by comparing with the standard curve of 2,2 diphenyl-1-picrylhydrazyl (DPPH) (Sadasivam, 1996). Methionine content was determined through hydrolysis of grain protein. In brief, grain protein was first hydrolyzed with mild acid, then methionine got liberated and gave a red color with nitroprusside under alkaline conditions. The resulting red color was further read spectrophotometrically at 520 nm, and then methionine content was calculated by using a standard curve (Sadasivam, 1996).

### 2.7 Soil samples analysis

Before sowing and at the stage of crop harvesting, the soil samples of every treatment were collected from each geographical location and soil collected from the same treatment of one location were pooled; the same process was done for the second location to make four composite soil samples (pre-sowing, post-harvest control, MP1 treated and N26 treated) from each location. In total, eight soil composites were prepared and subjected to various analytical and microbiological analyses to determine the effect of bioinoculants on available soil nutrient content, physio-chemical composition, and microbial community dynamics.

#### 2.7.1 Physio-chemical composition analysis

Soil samples collected from each location were subjected to soil physiochemical analysis. Soil pH was determined with the use of a pH meter by preparing soil suspension in distilled water (1:2.5 w/v). Soil organic carbon was calculated by employing the dichromate oxidation method (Walkley and Black, 1934). Available nitrogen in the soil was estimated as described by Subbiah and Asija (1956), whereas potassium content was determined through Jackson’s method (Jackson, 1967). Zn and Fe content of the soil was estimated through DTPA extraction method (Lindsay and Norvell, 1978). Further, to determine the dehydrogenase activity, the method of Casida et al. (1964) was followed, whereas total phosphatase activity was calculated by adding the acid and alkaline phosphatase activity. The method of Tabatabai and Bremner (1969) was used to determine the acid and alkaline phosphatase activity, which involves colorimetric estimation of p-nitrophenol released by phosphomonoesterases activity upon soil incubation with buffer at pH 6.5 for acid and 11 for alkaline phosphomonoesterases. Urease activity was measured through protocol as suggested by Bremner and Douglas (1971).

#### 2.7.2 Culturable diversity analysis

All collected soil samples were analyzed for culturable microbial diversity count through serial dilution method. Dilutions (1-10^8^) of each soil sample were prepared by taking 1 g of soil into 9mL of water blank following with transfer of 1mL in subsequent water blank; afterward, 1mL of soil suspension was poured into sterile Petri plate following with the addition of various nutrient medium (Nutrient agar, Potato dextrose agar, Actinomycetes agar, Pseudomonas agar, Azotobacter agar, Burk medium, Pikovskayas agar, Aleksandrow agar, Azospirillum agar) in separate Petri plate to support the growth of bacteria, fungi and actinomycetes. Afterward, each Petri plate was incubated at 25°C ± 2°C and regularly observed for the appeared microbial colonial growth. The number of appeared microbial colonies was counted through the colony counter, and CFU/g was calculated (Singh et al., 2020).

#### 2.7.3 Unculturable diversity analysis through next generation sequencing

To determine the native kidney bean rhizosphere diversity and microbial shift ensued from crop grown season and effect of treatments from presowing conditions, unculturable microbial diversity of the soil sample was analyzed through next-generation sequencing of the V3-V4 region of 16S rRNA gene. In brief, rhizospheric soil samples were collected from each plot (five different location of the plot) and soil collected from identical treatment at the same location got pooled for NGS. Subsequently, whole DNA present in all soil composite samples was extracted separately and aseptically through a Soil DNA isolation kit. Extracted DNA was quantified and quality was checked *via* NanoDrop at 260 nm to perform the PCR. The V3-V4 region of 16S rRNA gene was amplified through PCR. The amplified stretch of the DNA product was purified following the QC checks and library preparation of reads. Furthermore, illumine Miseq sequencing of the V3-V4 region of the 16S rRNA gene was performed with a 2x300PE v3 sequencing kit. The QC of sequenced reads was determined through multiqc. All the pre-processed sequences were subjected to trimming and chimera removal and then analyzed for OTU abundance through QIIME/MOTHUR/KRAKEN/BRACKEN workflow. Abundance of OTUs was determined and used for the classification, and unclassified sequences were termed as unknown OTUs. Afterward, alpha diversity was analyzed through various diversity indexes such as Chao1, Simpson, Shannon, and Fisher; further beta diversity was also investigated through principal coordinate analysis on the galaxy server. In addition, based on the microbial diversity present in the soil samples, the galaxy server was also used to determine the metabolic diversity predicted in the samples. Next generation sequencing was performed using Illumina Miseq at Biokart India Pvt. Ltd., Bangalore, India.

### 2.8 Statistical analysis

In order to test the significance and variance among treatments, analysis of variance (ANOVA) was performed. All agronomical, soil and nutritional quantitative data were subjected to one-way ANOVA by using R 3.6.1 at p < 0.005 level of significance. Further, Pearson’s correlation analysis was performed to determine the extent of plant yield and nutrient enhancement of the kidney bean by bacterial PGP potential. Moreover, cluster analysis was used to shape the multivariate data into subset. On the basis of hierarchical clustering a dendrogram was prepared to reveal the attributable effect of bacterial strains. Furthermore, principal component analysis was also performed to scrutinize the performance of strains under different altitudes, which simplifies the complexity of variable data on the basis of eigenvalue and determines the relative impact of different treatments on agronomical parameters.

## 3 Results

### 3.1 Plant growth promoting potential of bacterial strains

Bacterial strains used in the current study were already reported as potential phosphate solubilizers and atmospheric nitrogen fixers (Tomer et al., 2017; Rajwar et al., 2018; Joshi et al., 2019). Further, *in vitro* plant growth-promoting assessment of both cold adaptive bacterial strains (*Pseudomonas jesenii* MP1 and *Pseudomonas palleroniana* N26) confirmed that both have significant plant probiotic traits (**Table 1**). Phosphate solubilization efficiency of *Pseudomonas jesenii* MP1 and *Pseudomonas palleroniana* N26 ranged between 245.16 ± 8.6 µg/ml and 237.40 ± 6.7 µg/ml under the NBRIP broth medium. Further, AAS results demonstrated that *Pseudomonas palleroniana* N26 has the most tremendous zinc solubilization potential i.e. 19.34 ± 0.67 µg/ml, followed by *Pseudomonas jesenii* MP1, which has the ability to solubilized zinc up to 18.50 ± 0.55 µg/ml. In addition, *P. jesenii* MP1 and *P. palleroniana* N26 also showed iron chelation potential with a range of 42.55% and 33.94% siderophore unit, respectively, while utmost IAA production was demonstrated by *P. palleroniana* N26 (41.38 ± 1.26 µg/ml), followed by *P. jesenii* MP1 (28.03 ± 0.97 µg/ml). Both the bacterial strains showed EPS production, among them 4.17 ± 0.28 mg/ml EPS were produced by *P. jesenii* MP1, whereas, *P. palleroniana* N26 produced 3.33 ± 0.28 mg/ml. Further, both the bacterial strain also exhibited positive results for the qualitative biofilm and ammonia production test.

**Table 1:** Plant probiotic traits of *P. jesenii* MP1 and *P. palleroniana* N26.

SN	Name of Strain	Phosphate solubilization (µg/ml)	Solubilized zinc (µg/ml)	% siderophore unit	Solubilized potassium (µg/ml)	IAA production (µg/ml)	EPS Production (mg/ml)	Biofilm production	Ammonia production
1	*Pseudomonas jesenii* MP1	245.16 ± 8.6	18.50 ± 0.55	42.55 ± 1.05	122.33 ± 1.1	28.03 ± 0.97	4.17 ± 0.28	+	+
2	*Pseudomonas palleroniana* N26	237.40 ± 6.7	19.34 ± 0.67	33.94 ± 0.75	101.56 ± 2.7	41.38 ± 1.26	3.33 ± 0.28	+	+
SEM		7.92	0.509	0.907	8.94	1.09	0.273	NA	NA

Data were analyzed at P<0.05 level of significance. Mean ± SE is shown in the table; each value is the mean of four replicates.NA, not applicable. + = indicates presence of activity.

### 3.2 Yield and agronomical parameters

The prolific impact of both cold adaptive bacterial strains under field ambience was carried out at two different altitudinal locations i.e. Chakrata and Harsil, in Indian Central Himalayan regions. *P. jesenii* MP1 and *P. palleroniana* N26, both cold adaptive strains demonstrated a positive influence on agronomical and yield-associated parameters of kidney bean over uninoculated control at both the locations. Results demonstrated that the grain yield at the Harsil location is much higher than Chakrata location. This could be possible because Harsil located at 500 m higher elevation than Chakrata which results in high organic carbon and nutrient accumulation due to low decomposition rate (Schindlbacher et al., 2010). As the rainfall affects the yield (Kimani et al., 2022), the rainfall in Chakrata was bit lower than usual during the crop season which might affect the yield of kidney bean. Moreover, the local landrances of Harsil shows more yield potential compared to the landraces of Chakrata. The results of the study illustrated that agronomical parameters such as root, shoot and pod length, and number of grains per pod were highest for *Pseudomonas jesenii* MP1 primed seeds, whereas *P. palleroniana* N26 primed seeds showed the highest 1000 grain weight at the Harsil field. Further, higher grain yield i.e. 20.74% was observed among all treatments upon seeds priming with *P. jesenii* MP1 over uninoculated control at the Harsil site (**Table 2**). Similarly, at the Chakrata field site, *P. jesenii* MP1 and *P. palleroniana* N26 showed enhanced root, shoot, and pod length and number of grains per pod over uninoculated control. They also demonstrated at par average grain yield, i.e., 23.78%–23.34% compared to untreated control, indicating remarkable plant growth promotion potential of *P. jesenii* MP1 and *P. palleroniana* N26 under low temperature environmental conditions at higher altitudes (**Table 2**).

**Table 2:** Effect of *Pseudomonas jesenii* MP1 and *Pseudomonas palleroniana* N26 inoculation on growth and yield attributing characters of Kidney bean at Harsil and Chakrata.

Site	Treatments	Growth and yield attributing characters					
		Root length (cm)	Shoot length (cm)	Pod length (cm)	No. of grain/Pod	1000 grain weight (g)	Grain yield (q/ha)
Harsil	Control	8.9 ± 1.10^a^	94 ± 2.3^a^	10.76 ± 0.26^a^	4 ± 0.57^a^	23.27 ± 0.15^a^	7.23 ± 0.29^a^
	*Pseudomonas jesenii* MP1	11.70 ± 0.20^b^	116 ± 3.05^c^	11.73 ± 0.27^b^	5 ± 0.57^a^	24.85 ± 0.57^b^	8.73 ± 0. 35^c^ (20.74)
	*Pseudomonas palleroniana* N26	11.15 ± 0.12^b^	105 ± 2.64^b^	11.63 ± 0.38^b^	4.66 ± 0.33^a^	25.09 ± 0.52^b^	8.07 ± 0.39^b^ (11.61)
	SEM	0.53	3.4	0.21	0.29	0.36	0.22
Chakrata	Control	7.4 ± 0.48^a^	39.66 ± 3.52^a^	9.4 ± 0.58^a^	3.33 ± 0.33^a^	16.96 ± 0.19^a^	1.85 ± 0.06^a^
	*Pseudomonas jesenii* MP1	8.6 ± 0.39^b^	44 ± 3.05^a^	11.2 ± 0.23^b^	4 ± 0.0^a^	17.85 ± 0.32^b^	2.29 ± 0.09^b^ (23.78)
	*Pseudomonas palleroniana* N26	8.13 ± 0.17^b^	45 ± 2.30^a^	10.93 ± 0.12^b^	4 ± 0.0^a^	19.29 ± 0.37^c^	2.28 ± 0.09^b^ (23.24)
	SEM	0.25	1.7	0.33	0.14	0.37	0.05

Data were analyzed at P<0.05 level of significance. Mean ± SE is shown in the table; each value is the mean of eighteen replicates. Values in brackets indicate percent increase over control.Same superscripted letters in same column demonstrate insignificant whereas different superscripted letters demonstrate significant difference among the treatments.

### 3.3 Nutritional status of seeds samples

The nutrient content of seeds determines their quality because it is the only part humans consume to meet their nutritional needs. In order to assess the effect of cold adaptive bacterial strains on the nutritional status of seeds, protein, carbohydrate, antioxidant activity, methionine content, zinc, and iron content were estimated in seeds samples. The current study results demonstrated that proficient PGPR strains not only enhanced the plant’s growth and yield over uninoculated control but also augmented the seed’s nutritional status at both locations. The highest protein content i.e. 22.89% ± 3.8% and 25.34% ± 2.9% was recorded in seeds primed with *P. palleroniana* N26, which is closely followed by *P. jesenii* MP1 primed seeds at both locations (**Table 3**). In addition, uppermost carbohydrate content (i.e., 68.35 ± 1.60 and 66.40 ± 2.96) was also observed in seeds upon *Pseudomonas palleroniana* N26 priming at both locations, whereas *P. jesenii* MP1 primed seeds demonstrated the highest methionine content i.e., 0.0463 and 0.035 at Harsil and Chakrata, respectively. Further, seeds primed with proficient PGPR showed lower antioxidant activity over uninoculated control. The results of both sites revealed that maximum Zn and Fe content was reported in seeds primed with *Pseudomonas jesenii* MP1 at Harsil and *Pseudomonas palleroniana* N26 primed seeds at Chakrata (**Table 3**). These results indicated that both PGPR has a prolific effect on the nutritional status of seeds.

**Table 3:** Effect of *Pseudomonas jesenii* MP1 and *Pseudomonas palleroniana* N26 inoculation on nutrient status of Kidney bean at Harsil and Chakrata.

Sites	Seed sample with treatment	Protein content (%)	Carbohydrate content (mg/100g)	Methionine content (mg/g)	Antioxidant activity (mgAAE/g)	Zn content (mg/kg)	Fe content (mg/kg)
Harsil	Untreated Control	18.39 ± 4.2^a^	65.76 ± 2.8^a^	0.043 ± 0.0025^a^	1.08 ± 0.15^a^	41.16 ± 4.0^a^	34.83 ± 3.4^a^
	*Pseudomonas jesenii* MP1 treated seeds	20.39 ± 1.4^a^	67.26 ± 1.43^a^	0.046 ± 0.0008^a^	1.02 ± 0.04^a^	53.66 ± 3.7^b^	39.7 ± 1.17^a^
	*Pseudomonas palleroniana* N26 treated seeds	22.89 ± 3.8^a^	68.35 ± 1.60^a^	0.046 ± 0.0051^a^	1.00 ± 0.02^a^	50.16 ± 0.6^b^	36.83 ± 1.48^a^
	SEM	1.77	1.11	0.001	0.05	2.45	1.34
Chakrata	Untreated Control	21.99 ± 2.2^a^	65.81 ± 1.57^a^	0.025 ± 0.0060^a^	0.815 ± 0.29^a^	28.33 ± 3.21^a^	56.00 ± 2.5^a^
	*Pseudomonas jesenii* MP1 treated seeds	23.34 ± 3.2^a^	66.36 ± 0.46^a^	0.035 ± 0.0057^a^	0.71 ± 0.16^a^	41.83 ± 1.5^b^	65.83 ± 3.03^b^
	*Pseudomonas palleroniana* N26 treated seeds	25.34 ± 2.9^a^	66.40 ± 2.96^a^	0.027 ± 0.0036^a^	0.47 ± 015^a^	45.66 ± 1.6^b^	79.16 ± 5.84^b^
	SEM	1.26	0.83	0.002	0.11	2.86	3.92

Data were analyzed at P<0.05 level of significance. Mean ± SE is shown in the table; each value is the mean of four replicates.Same superscripted letters in same column demonstrate insignificant whereas different superscripted letters demonstrate significant difference among the treatments.

### 3.4 Soil health analysis

Soil is the reservoir of nutrients which supports plant growth and development. Hence, the amount of nutrients and its holding capacity determine soil health. Thus, the nutritional assessment was done through various physiochemical and enzymatic analysis of soil collected before sowing and at harvesting stage from both locations. The soil nutrient and enzyme status of harvesting stage soil was remarkably improved upon cold adaptive bioinoculants treatment. On the other side, a minute difference was observed among the soil pH of all the samples collected from Harsil and Chakrata sites. Among all treatments, *P. jesenii* MP1 treated soil of Harsil site exhibited the highest available macro and micronutrient content, whereas in Chakrata, *P. palleroniana* N26 treated soil exhibited the highest available nitrogen and potassium content, but maximum available micronutrients were again found in *P. jesenii* MP 1 treated soil (**Tables 4A**, **4B**). The highest activity of soil enzymes was found in *P. palleroniana* N26 treatment at both locations.

**Table 4A:** Effect of potential bioinoculants on soil physio-chemical properties of kidney bean at farmer’s field of Chakrata.

CHAKRATA										
			Available macro-nutrients (Kg/ha)		Available micro-nutrients (µg/mg)			Soil Enzymes		
Treatments	pH	Organic carbon (%)	Nitrogen	Potassium	Zinc	Iron	Copper	Dehydrogenase µg TPF/h/g soil	Total phosphatase µg p-nitro/g soil/h	Urease µg urea/g soil/h
Presowing	7.5 ± 0.0^a^	1.24 ± 0.04^a^	208.23 ± 3.0^a^	572 ± 2.64^a^	1.217 ± 0.05^C^	39.92 ± 0.6^b^	0.835 ± 0.00^C^	132.68 ± 1.5^a^	244.42 ± 6.0^a^	29.49 ± 0.3^a^
Control	7.6 ± 0.1^a^	1.25 ± 0.05^a^	219.52 ± 5.5^b^	700 ± 12.7^b^	0.746 ± 0.00^a^	28.73 ± 0.5^a^	0.356 ± 0.00^a^	155.80 ± 2.4^b^	265.01 ± 3.2^b^	31.67 ± 0.4^b^
Pseudomonas jesenii MP1 treated soil	7.5 ± 0.0^a^	1.41 ± 0.01^b^	238.34 ± 1.8^d^	725 ± 4.04^c^	1.242 ± 0.00^C^	49.80 ± 0.3^c^	1.039 ± 0.00^d^	174.19 ± 1.1^c^	344.54 ± 9.9^d^	33.85 ± 0.7^c^
Pseudomonas palleroniana N26 treated soil	7.5 ± 0.0^a^	1.19 ± 0.01^a^	229.56 ± 4.0^c^	707 ± 4.04^d^	1.121 ± 0.02^b^	46.17 ± 0.0^c^	0.713 ± 0.00^b^	239.25 ± 4.9^d^	285.60 ± 5.7^c^	35.51 ± 0.8^d^
SEM	0.060	0.039	4.053	4.819	0.027	0.274	0.007	2.450	4.083	0.559

Data were analyzed at P<0.05 level of significance. Mean ± SE is shown in the table; each value is the mean of four replicates.Same superscripted letters in same column demonstrate insignificant whereas different superscripted letters demonstrate significant difference among the treatments.

**Table 4B:** Effect of potential bioinoculants on soil physiochemical properties of kidney bean at farmer’s field of Harsil.

Harsil										
			Available macro-nutrients (kg/ha)		Available micro-nutrients (µg/mg)			Soil Enzymes		
Treatments	pH	Organic carbon (%)	Nitrogen	Potassium	Zinc	Iron	Copper	Dehydrogenase µg TPF/h/g soil	Total phosphatase µg p-nitro/g soil/h	Urease µg urea/g soil/h
Presowing	7.3 ± 0.05^a^	0.81 ± 0.017^a^	224.54 ± 3.05^a^	727 ± 4.04^bc^	0.892 ± 0.009^b^	34.80 ± 0.972^b^	0.428 ± 0.013^b^	53.50 ± 0.45^a^	170.76 ± 1.55^a^	32.18 ± 0.68^a^
Control	7.4 ± 0.11^a^	0.75 ± 0.023^a^	252.13 ± 2.0^b^	678 ± 3.21^a^	0.825 ± 0.005^a^	31.76 ± 0.347^a^	0.346 ± 0.003^a^	82.17 ± 1.55^b^	207.78 ± 1.60^b^	32.56 ± 0.61^a^
Pseudomonas jesenii MP1 treated soil	7.6 ± 0.10^a^	1.12 ± 0.015^b^	284.75 ± 1.35^c^	714 ± 6.11^b^	1.063 ± 0.009^c^	42.54 ± 0.682^c^	0.978 ± 0.007^d^	150.22 ± 1.15^c^	223.64 ± 1.75^c^	37.18 ± 0.68^b^
Pseudomonas palleroniana N26 treated soil	7.6 ± 0.15^a^	1.27 ± 0.041^C^	294.78 ± 2.37^d^	742 ± 5.68^c^	0.817 ± 0.006^a^	34.98 ± 0.061^b^	0.540 ± 0.011^c^	276.91 ± 4.85^d^	276.91 ± 3.66^d^	38.60 ± 0.11^b^
SEM	0.096	0.092	2.584	3.215	0.009	0.482	0.010	2.545	1.952	0.403

Data were analyzed at P<0.05 level of significance. Mean ± SE is shown in the table; each value is the mean of four replicates.Same superscripted letters in same column demonstrate insignificant whereas different superscripted letters demonstrate significant difference among the treatments.

### 3.5 Pearson’s correlation coefficient

Correlation among PGP traits of both bioinoculants and nutritional status of bioinoculants primed seeds was assessed. The results of the correlation revealed that phosphate and potassium solubilization have a positive correlation with methionine content, whereas zinc solubilization and IAA production has positive attribute for protein, carbohydrate, zinc, and iron content. Further, siderophore production showed a positive correlation with methionine, zinc, and iron content and negative for protein and carbohydrate content (**Table 5**). It indicates that PGP properties of bioinoculants are involved in nutrient augmentation and prominently improve the nutrients in kidney bean grains.

**Table 5:** correlation among PGP traits of both bioinoculants and nutritional status.

	Phosphate solubilization (µg/ml)	Solubilized zinc (µg/ml)	% siderophore unit	Solubilized Potassium (µg/ml)	IAA production (µg/ml)	Protein content (%)	Carbohydrate content	Methionine content (mg/g)	Zn content	Fe content (mg/kg)
Phosphate solubilization (µg/ml)		1.00E-10	1.00E-10	1.00E-10	1.00E-10	0.36143	0.69065	0.75049	0.98157	0.85293
Solubilized zinc (µg/ml)	-1		1.00E-10	1.00E-10	1.00E-10	0.36143	0.69065	0.75049	0.98157	0.85293
% siderophore unit	1	-1		1.00E-10	1.00E-10	0.36143	0.69065	0.75049	0.98157	0.85293
Solubilized Potassium (µg/ml)	1	-1	1		1.00E-10	0.36143	0.69065	0.75049	0.98157	0.85293
IAA production (µg/ml)	-1	1	-1	-1		0.36143	0.69065	0.75049	0.98157	0.85293
Protein content (%)	-0.63857	0.63857	-0.63857	-0.63857	0.63857		0.54312	0.14138	0.28375	0.18466
Carbohydrate content	-0.30935	0.30935	-0.30935	-0.30935	0.30935	-0.45688		0.16199	0.32754	0.11401
Methionine content (mg/g)	0.24951	-0.24951	0.24951	0.24951	-0.24951	-0.85862	0.83801		0.25328	0.005845
Zn content	-0.01843	0.018433	0.01843	-0.01843	0.018433	-0.71625	0.67246	0.74672		0.21798
Fe content (mg/kg)	-0.14707	0.14707	0.14707	-0.14707	0.14707	0.81534	-0.88599	-0.99416	0.78202	

### 3.6 Cluster and principal component analysis

**Figure 1:**
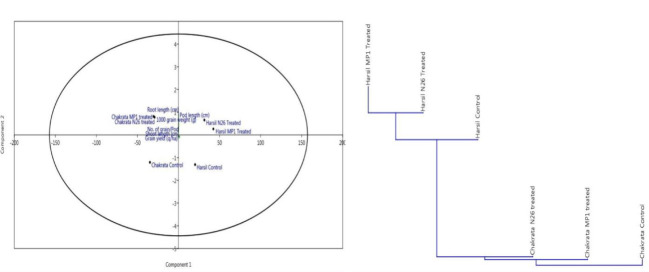
Principal component and cluster analysis depicting the effect of bioinoculants on agronomical parameters and their relative performance among treatments.

In order to assess the comparative prolific effect of both bioinoculants on agronomical parameters at different locations, a principal component analysis was performed. The results of the principal component analysis revealed that both bioinoculants have a positive effect on agronomical and yield related parameters at both locations, but bioinoculants *P. jesenii* MP1 and *P. palleroniana* N26 have demonstrated the most promising results at Harsil location. In addition, both bioinoculants *P. jesenii* MP1 and *P. palleroniana* N26 have clustered together over uninoculated control. Hence, the outcome of clustering also confirms the prolific effect of bioinoculants over control (**Figure 1**).

### 3.7 Culturable microbial diversity

**Figure 2:**
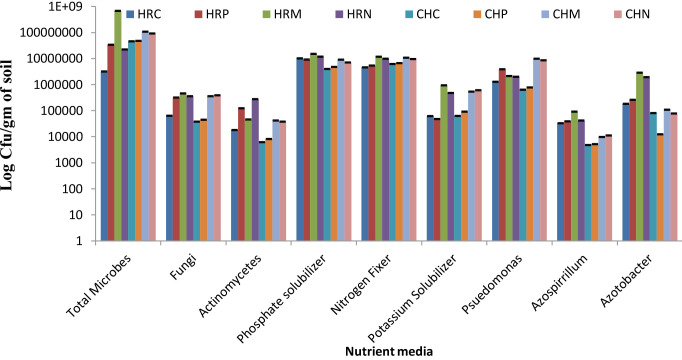
Culturable bacterial counts (cfu/g dry weight soil) of Chakrata and Harsil soils on different nutrient media. HRP, Harsil Pre-sowing Soil, HRC, Harsil control soil, HRM, Harsil MP1 treated soil, HRN, Harsil N26 Treated soil, CHP, Chakrata Pre-sowing soil, CHC, Chakrata control soil, CHM, Chakrata MP1 treated soil, CHN, Chakrata N26 Treated Soil.

The total culturable microbial count was measured on different mediums to assess the more profound view of microbial diversity present and altered microbial shift in soil. Results of the culturable counts revealed that both bioinoculants treatment supports the growth of nutrient solubilizing microbial community in the rhizosphere demonstrating the superfluous microbial population over pre-sowing and control conditions (**Figure 2**). Among both bioinoculants, the treatment of *P. jesenii* MP1 significantly attracted the nutrient-solubilizing microbial community, hence demonstrating the highest CFU/g in almost all the mediums. Further results also reveal that Harsil soil has lower bacterial abundance than the Chakrata soil but contains more fungi and actinomycetes. Further, almost similar pool of phosphate, nitrogen and potassium solubilizers were present in the soil of Harsil and Chakrata. In addition, a high number of *Pseudomonas*, *Azospirillu*m, and *Azotobacter* were found in the soil of Harsil compared to the Chakrata soil (**Figure 2**).

### 3.8 Unculturable microbial diversity

**Figure 3:**
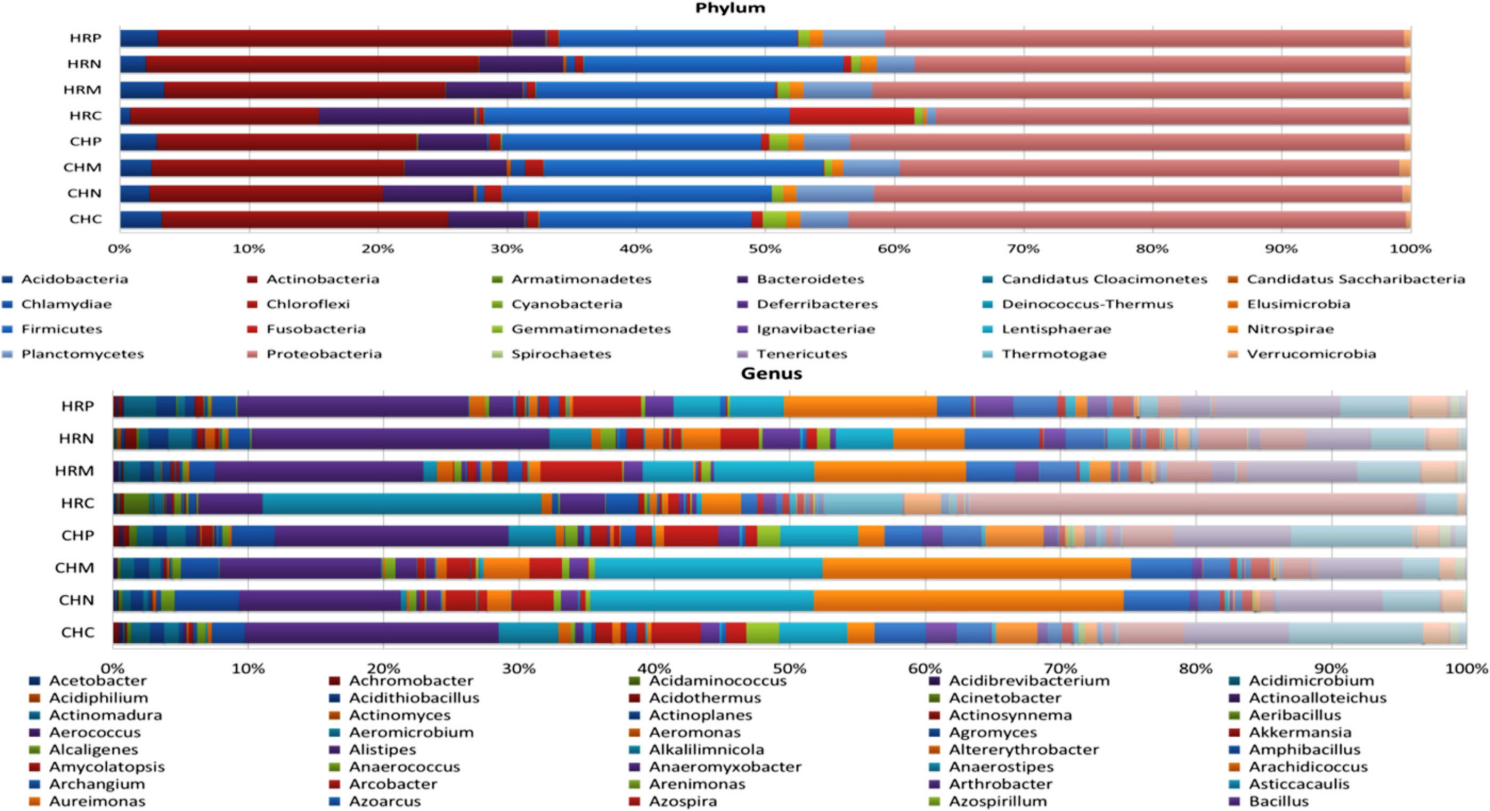
Stacked bar plot representing relative abundance; **(A)** Phylum and **(B)** Genus distribution among treatment and control under different locations. Where, HRP = Harsil Pre-sowing Soil, HRC = Harsil control soil, HRM = Harsil MP1 treated soil, HRN = Harsil N26 Treated soil, CHP = Chakrata Pre-sowing soil, CHC = Chakrata control soil, CHM = Chakrata MP1 treated soil, CHN = Chakrata N26 Treated Soil.

**Figure 4:**
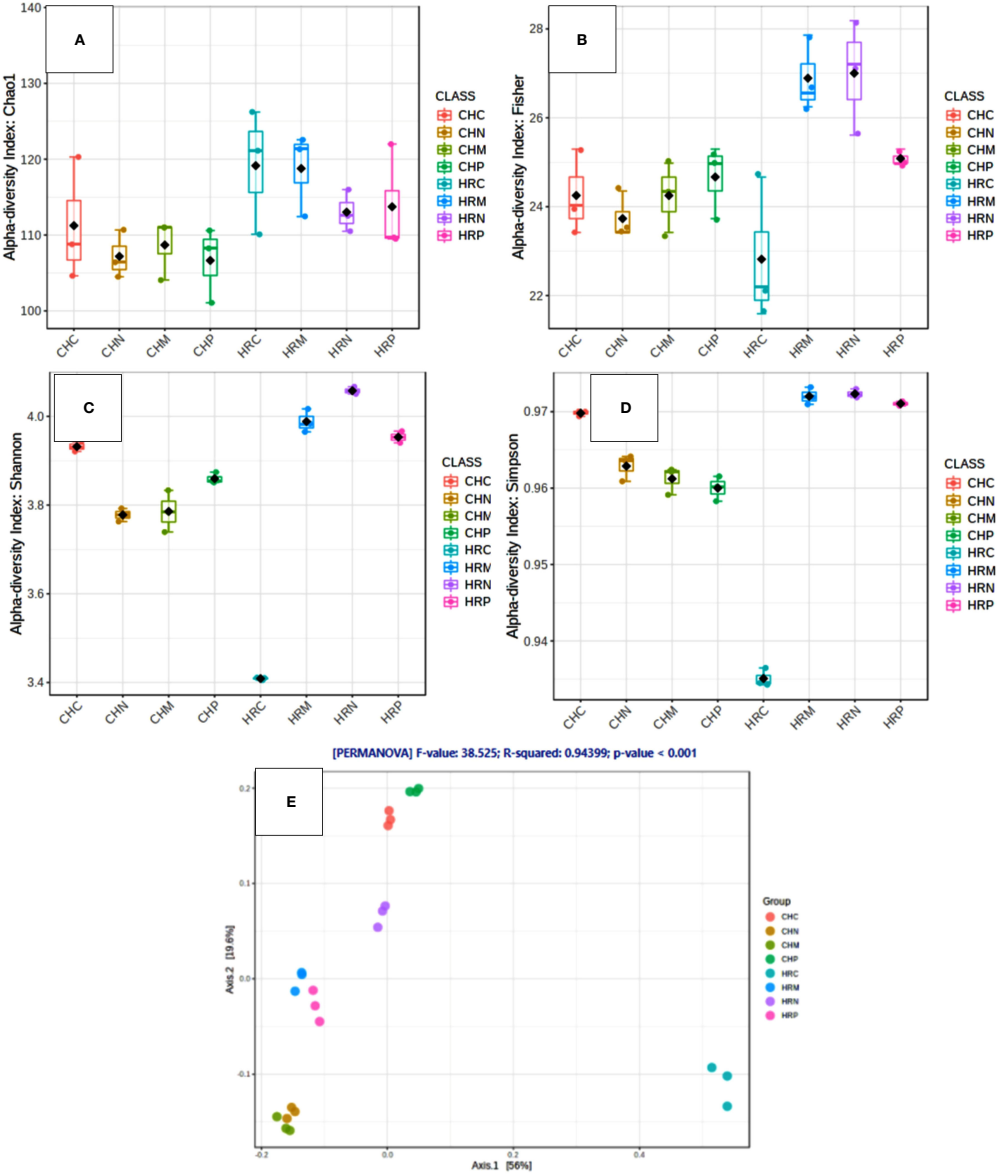
Diversity analysis within and among the samples through various indices. **(A–D)** Alpha Diversity through **(A)** Chao1 **(B)** Fischer **(C)** Shannon **(D)** Simpson and beta diversity through **(E)** principal coordinate analysis.

Unculturable microbial composition present in the soil samples was determined through metagenomic analysis through next-generation sequencing. A sum of around 26 lakh amplicons was retrieved from 16S rRNA gene amplicon sequencing. These amplicons were allocated to 66376.00 OTUs. Representative OTUs revealed diverse colonization of bacterial flora among different cultivation locations, i.e., Harsil and Chakrata. At both the field sites, the highest populated OTUs belong to the phylum Proteobacteria, Firmicutes, Actinobacteria, Acidobacteria, etc. However, the domination of OTUs of these phyla was more in rhizospheric soil of Harsil, compared to Chakrata soil. In regard to specific location, pre-sowing soil of Chakrata was highly populated with bacteria of 15 different phyla and control was dominated by 16 different phyla, whereas *P. jesenii* MP1 and *P. palleroniana* N26 treated soil was dominated by 18 and 15 bacterial phyla, respectively (**Figure 3**). In addition, at the Harsil site pre-sowing soil was dominated by 17 different phyla; however, *P. jesenii* MP1 and *P. palleroniana* N26 treated soil with 20 and 19 bacterial phyla. These phyla are further diversified into 52 classes, 107 orders, 197 families, and 343 genera. Among them, Harsil pre-sowing soil, soil treated with *P. jesenii* MP1 and *P. palleroniana* N26 were predominated with Genus *Bacillus* with 15%, 22%, and 15%, respectively, while the uninoculated soil of Harsil prevailed with *Fusobacterium* (28%) and *Bacillus* (8%). On the other side, Chakrata pre-sowing soil was dominated with *Lactobacillus* (29%) and uninoculated soil with *Bacillus* (17%), whereas *P. jesenii* MP1- and *P. palleroniana* N26-treated soil was predominated with *Clostridioides* (20%, 21% respectively) and *Bacillus* (11%). A massive number of OTUs were unassigned at each step of classification, indicating the presence of unique microbial diversity in Chakrata and Harsil soils. Further, in order to determine the microbial diversity and abundance among treatments, alpha diversity through Chao1, Shannon, Simpson, and Fisher and beta diversity through PCA was analyzed (**Figure 4**). Simpson index revealed that at Harsil and Chakrata *P. jesenii* MP1 and *P. palleroniana* N26 treated soil have almost the same abundant species, whereas Harsil control soil has low species abundance, but Chakrata control soil has more abundance. In addition, the Shannon index demonstrated that *P. jesenii* MP1 and *P. palleroniana* N26 treated soil of Harsil is highly diversified compared to control soil, whereas in Chakrata control soil was more diversified than treated ones. Further alpha diversity analysis through Chao1 and Fisher revealed higher species diversity in *P. jesenii* MP1 and *P. palleroniana* N26 treated soil samples of Harsil over pre-sowing and uninoculated samples, whereas species diversity was almost the same among all samples of Chakrata. Further, the PCA chart demonstrated the clustering of Chakrata control and pre-sowing soil, *P. jesenii* MP1- and *P. palleroniana* N26-treated soil; hence, they must sustain similar kinds of microorganisms. Similarly, pre-sowing and control soil of Harsil must sustain similar microorganisms because they clustered together, whereas *P. jesenii* MP1- and *P. palleroniana* N26-treated soil did not cluster together in chart hence did not upholds similar bacterial population.

**Figure 5:**
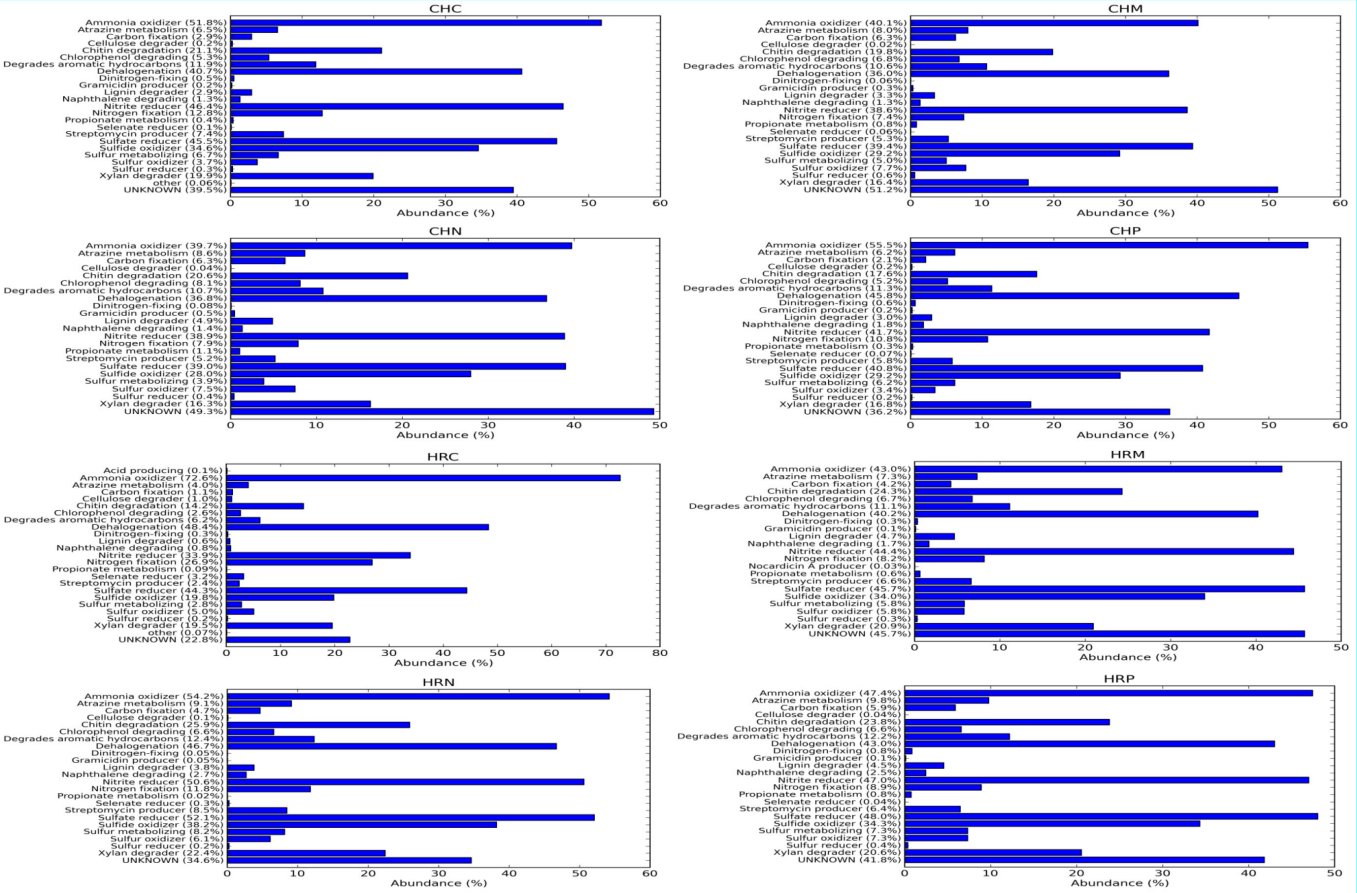
Metabolic functional annotation and amplitude of metabolic pathways governed by native bacterial diversity among treatment and control under different locations.

Afterward, metabolic diversity was also predicted on the basis of 16S rRNA gene diversity through Picrust (**Figure 5**). Results of metabolic diversity analysis revealed that at Harsil site, the amplitude of carbon fixation, Chitin and xylan degrader, atrazine metabolism, streptomycin producer was high and ratio of ammonia oxidizer *vs*. nitrite reducer and nitrogen fixation was low in *P. jesenii* MP1 and *P. palleroniana* N26-treated soil as compared to uninoculated soil. Further, at the Chakrata site, carbon fixation, atrazine metabolism, streptomycin producer, and ratio of ammonia oxidizer *vs*. nitrite reducer were high in *P. jesenii* MP1- and *P. palleroniana* N26-treated soil as compared to uninoculated soil. In addition, a high percentage of unknown metabolic activity was reflected in *P. jesenii* MP1- and *P. palleroniana* N26-treated soil of Chakrata (51% and 49%, respectively) and Harsil (45.8% and 34.6%, respectively).

## 4 Discussion

After the green revolution, agriculture production was boosted with increased fertilizer application, high-yielding varieties, etc. But in recent years, yield and nutrients are not mounting proportionally to the agrochemical input. Though, it is deteriorating for soil health and biodiversity (Khan et al., 2020b). In the present scenario, the feeding of the ever-growing world’s population sustainably needs the acceleration of food production. In order to accelerate agriculture production in the hill region without harming the soil and the environment, we must shift toward sustainable and effective practices like applying potential bio inoculants. The kidney bean is a highly economical cash crop in the Indian central Himalayan region, requiring excess phosphorus and nitrogen for optimum growth (Kakon et al., 2016). Fragmented lands, low nutrient accessibility, and poor agronomical practices carried by the hill farmers are the major reason for reduced kidney bean yield and nutrient content (Kumar et al., 2021). Addressing such problem, the current study reveals the credibility of potential cold adaptive potent bioinoculant application, i.e., *P. jesenii* MP1 and *P. palleroniana* N26, in improving yield, nutrient status, and soil health in hill regions. Both bioinoculants used in this study were segregated from Himalayan soil and formerly characterized for their phosphorous solubilization and nitrogen fixating ability (Tomer et al., 2017; Rajwar et al., 2018; Joshi et al., 2019). During the research, the extended plant growth promoting potentialities of both bioinoculants were revealed and having promising phosphate, zinc, and potassium solubilization potential with the decline in broth pH. This dropping of pH signifies organic acid production in broth, which is considered essential for P, Zn, and K solubilization (Upadhayay et al., 2022b). The accretion of gluconic acid, oxalic acid, formic acid, tartaric acid, acetic acid, lactic acid, citric acid, malonic acid etc. has been observed during P, Zn, and K solubilizing broth medium with a decline of pH, which might aid in solubilization of the desired nutrient from their insoluble sources (Fasim et al., 2002; Li et al., 2010; Mumtaz et al., 2017; Vidyashree et al., 2018; Othman et al., 2022). Moreover, Ramesh et al. (2014) also found that the decline in pH and solubility of complex zinc is correlated with organic acid production. A study by El-Sayed et al. (2014) agrees with the present study, which also reported the efficient P, Zn, and K solubilization from insoluble zinc sources with the decline in pH and further concluded the auxiliary role of organic acid production in P, Zn, and K solubilization. Furthermore, both bacterial strains also have been reported to produce varying siderophore production, which has a role in metals (such as iron) capturing and making accessible for plants. This microbial-assisted iron supply improves chlorophyll and photosynthesis; furthermore, it also augments the iron accumulation in the edible parts (Kroh and Pilon, 2020; Sun et al., 2021). Similarly, in the present study both bioinoculants have siderophore production ability which has assisted in the microbial mediated iron accumulation in the kidney bean grains. Similarly, Rahimi et al. (2020) also reported siderophore production in the bacterial strain (*P. Fluorescens* and *M. Yunnanensis*) and reported augmented chlorophyll and iron content upon bacterial priming under iron deficient conditions. In addition, siderophore also augments zinc solubilization (Kamran et al., 2017), which was also confirmed by correlation analysis, as % siderophore showed a positive correlation with Zn content in grains. Besides, now it is an established fact that IAA production by microbes is a direct plant growth promontory mechanism, as IAA assists in plant growth, root proliferation, tissue differentiation, elongation, embryonic development, etc. (Egamberdieva, 2011; Cheng et al., 2022). In the present study, a considerable amount of IAA production was reported by both i.e., *P. jesenii* MP1 and *P. palleroniana* N26 cold adaptive bioinoculants ranging from 28.03 ± 0.97 and 41.38 ± 1.26 µg/ml, respectively. Previously, IAA production ranging from 7.97 and 134.67 µg/ml was also reported by Bhattacharyya et al. (2020) in different bacterial strains and concluded its plant growth potential. Both the cold adaptive bioinoculants used in this study also had the EPS and biofilm producing capability, which is responsible to concentrate the nutrient in the rhizosphere for root uptake. EPS and biofilm are hygroscopic. Therefore, they facilitate soil aggregation and retain moisture (Costa et al., 2018; Keuschnig et al., 2022). Moreover, it imparts effective colonization of bioinoculants around the root and acts as the nutrient sink for uptake; simultaneously, it also challenges pathogen colonization by covering root binding sites (Bhagat et al., 2021). Similar results of EPS and biofilm formation were also reported by Haque et al. (2020) and they observed augmented plant growth and nutrients upon EPS and biofilm producing bioinoculants. Both bioinoculants were also tested for ammonia production. This feature is attributable to nitrogen supply, which is crucially required for kidney bean productivity (Basnet et al., 2022). Alori and Babalola (2018) documented the role of ammonia-producing microbes in nitrogen supply augmentation for improved plant growth and productivity. Results of the present investigation also revealed the prolific influence of cold adaptive bioinoculant application on agronomical parameters like root, shoot, pod length, number of grains per pod, weight of 1000 grains, and yield over uninoculated control at both locations. Similarly, Singh et al. (2018) and Mekonnen et al. (2022) also reported the improved agronomical parameters of lentil and pea crop, respectively, upon potent bioinoculant application. Protein and carbohydrates are the two out of three main nutrients of any diet and are primarily involved in energy metabolism and growth. Further, methionine is an amino acid and takes part in protein synthesis and also acts as precursor for other amino acids like cysteine, etc. (Brosnan and Brosnan, 2006). Under both the locations, bioinoculant application has enhanced the protein, carbohydrate, and methionine content in seed samples. Therefore, it can be concluded that *P. jesenii* MP1 and *P. palleroniana* N26 bioinoculant application augmented the yield and grain’s nutritional status of kidney bean. The present study agreed with Pandey et al. (2018) who reported the enhanced protein, carbohydrate, and methionine content in *Amaranthus hypochondriac* grains upon bioinoculant application. The “microbial assisted biofortification” is a novel practice to fortify grains with micronutrients such as Zn, Fe, Mn, etc. Zinc is an essential element as it takes part in IAA production, acts as cofactor for enzymes, and is necessary for fertilization. On the other side, iron is involved in energy metabolism (Upadhayay et al., 2022c). The results of the current study demonstrated a remarkable increment in zinc and iron content of grains upon bioinoculant application at both locations, which also confirms the biofortification ability of cold adaptive potential bioinoculants under field conditions. Similar findings were also reported by Hussain et al. (2020), which confirmed enhanced zinc and iron content when different PGPRs were applied to wheat seeds. Nutrients are part of the soil, but their accessibility could be a question for healthier soil. A healthier soil must sustain accessible nutrients, and then only it can support the plant’s functioning and growth. The current study’s findings demonstrated that both bioinoculants improved macro and micronutrients over uninoculated control soil at both locations, which is also the sole reason for the kidney bean crop’s improved growth and agronomical attributes. The enhancement of macro and micronutrients inside the soil might be due to the nutrient solubilization potential of the bioinoculants. Cig et al. (2021) also agree with the present finding, which also reported improved the macro and micronutrient content and enhanced the yield of wheat crops upon *Bacillus subtilis* and *Paenibacillus azotofixans* inoculation. Soil nutrients and enzymes are interconnected because enzymes regulate macro and micronutrient content and are considered soil health indicators. The current finding confirms that a significant increment in total phosphatase, dehydrogenase, and urease activity was observed in bioinoculant-treated samples compared to control at both locations. Total phosphatase is involved in removing phosphate molecule from its insoluble molecule; dehydrogenate enzyme denotes the redox potential of soil, as it takes part in the oxidation of soil organic matter, further urease degrades the urea present in the soil and contributes into available nitrogen (Sherene, 2017; Bogati and Walczak, 2022). Improved soil enzyme activity is due to bioinoculant application, considered beneficial for nutrient mobilization, native microflora, and soil health. Augmentation of soil enzymes and nutrients are also attributable to enhanced microbial activity, which is reflected in the results of the current investigation, which were confirmed through the significant increment in total microbial activity and nutrient mobilizers upon bioinoculant application. An elevated level of microorganisms and nutrient solubilizers also caused improved nutrients in the soil and subsequently flourished plant growth and productivity. Similar to the present study, Chaudhary et al. (2021) also concluded the enhanced microbial activity attributable to improved plant growth and soil nutrients and health. The hilly soil encompasses enormous diversity, but its composition depends upon native environmental conditions and altitudinal variation. Metagenomic analysis of soil revealed the proliferation of highly diversified bacterial communities at both locations as they possess enormous microbes of different taxa. In addition to this, numerous sequences were found to be unassigned OTUs in all samples, which can be correlated with the persistence of novel microbes in the present study. All the samples from both locations demonstrated microbe dominance that belongs to the phylum Proteobacteria, Firmicutes, Actinobacteria, Acidobacteria, etc. A high ratio of Proteobacteria and Actinobacteria reflects the premium quality of soil (Wu et al., 2019), which is reported in the current study. Further, the domination of OTUs of these phyla was much more in the rhizospheric soil of Harsil compared to Chakrata soil. This might be due to the selection pressure of the high altitude of Chakrata. The dominance of OTUs of phylum Proteobacteria indicates a favorable plant growth environment as its OTUs constitute numerous plant growth promoters (Lugtenberg and Kamilova, 2009; Dai et al., 2018). In addition, *Acidobacteria* is also attributable to organic carbon availability and disease suppression (Ayangbenro et al., 2022). Further, at the Harsil site, *P. jesenii* MP1 and *P. palleroniana* N26 inoculation shifted the microbiota with percentage increment of *Bacillus* over uninoculated control (8% to 15%–21%), which is known for its massive plant growth promoting potency (Hashmi et al., 2020). Further, members of Firmicutes, Planctomycetes, Nitrospirae, Gemmatimonadetes, and Cyanobacteria, were also dominated in all samples. The presence of members of Nitrospirae also confirms the presence of nitrogen transformation as these bacteria are cold adaptive nitrite-oxidizing bacteria hence they convert nitrite into nitrate (Dasauni and Nailwal, 2020).

Concerning the diversity and abundance of the microbial community, Shannon, Chao1, Fisher, and Simpson indexes of alpha diversity reflected increment in diversity and abundance of the microbial community upon *P. jesenii* MP1 and *P. palleroniana* N26 inoculation over control at the Harsil site whereas at the Chakrata site diversity and abundance was at par with control soil. Between the two locations, Harsil soil was found with more richness and abundance over Chakrata. Kimani et al. (2022) reported the reduction in kidney bean yield due to the reduction in rainfall. Similarly, the average rainfall was a bit lower in the Chakarata region than usual, which might be the reason for reduced microbial abundance in Chakrata. Whereas, the flourished microbial community due to the optimum rainfall at Harsil could be the reason for better growth and yield of kidney bean at the Harsil location. Similar to the present study, increased diversity, and microbial community abundance were also reported by Chaudhary et al. (2021) upon nanogypsum and *P. Taiwanensis* inoculation with maize plants. Further, metabolic diversity prediction in the current study also revealed that the amplitude of carbon fixation, chitin, and xylan degrader, atrazine metabolism, and streptomycin producer was enhanced due to *P. jesenii* MP1 and *P. palleroniana* N26 treatment in the soil as compared to the uninoculated control, which can be correlated with augmented carbon reserve, nutrient acquisition from insoluble sources, and pathogen protection in the treated soil. In addition, a high percentage of unknown metabolic activity was reflected in *P. jesenii* MP1- and *P. palleroniana* N26-treated soil of Chakrata (51% and 49%) and Harsil (45.8% and 34.6%), suggesting induction of novel functions beneficial for plant growth and soil activity. Further, a correlation study also revealed that the PGP traits of bacterial strains are attributable to nutrient enhancement, which also confirms the potential of bioinoculants in considerable improvement in the nutrients of kidney bean grains. In addition, PCA and clustering analysis outcomes revealed the prolific effect of bioinoculants on kidney bean yield at both locations. A similar study by Qi et al. (2021) also determined the best treatment through PCA and the role of plant growth promoting property attributable to plant physiological parameters through correlation analysis. Similar PCA cum clustering method was also employed by Sharma et al. (2011) for determining the best *Pseudomonas* spp. strain having best impact on total system productivity and nutrient status. The statistical analysis of the present findings confirms that both bioinoculants have massive credibility for yield, nutrients status, and soil health augmentation in hill regions under low temperature conditions. Hence, the bioinoculants *P. jesenii* MP1 and *P. palleroniana* N26 could be processed and exploited as commercial bioinoculants in the Central Himalayan region to improve the kidney bean yield and farmer’s income.

## 5 Conclusion

Hilly areas consist of different soil types and qualities. Altitudinal variation and cold temperatures contribute to soil texture and fertility, which modulates the soil biological processes, plant growth, and productivity accordingly. The kidney bean is a widely grown crop in the hilly region, but its production and quality are diminishing due to poor agronomic practices and low nutrient accessibility. The present study was undertaken to conquer such problem and to illustrate the plant growth promoting potential of cold adaptive *Pseudomonas jesenii* MP1 and *Pseudomonas palleroniana* N26 under low-temperature conditions at higher altitudes. The outcome of field trials in both locations revealed the conspicuous effect of bioinoculants in terms of growth and yield. Both strains acted as potential nutrifying agents as they also improved the nutrient content in grains as well. These strains not only augmented yield and nutrients but also improved soil health and replenished microbial diversity and abundance. The above results suggest that both bioinoculants are not only potent plant growth promoters but also preserve the environment and maintain sustainability; hence, these bioinoculants could be used as a green technology for yield enhancement and biofortification of the kidney bean in hilly regions.

## Data availability statement

The original contributions presented in the study are publicly available. This data can be found here: NCBI, PRJNA891453 and PRJNA890028.

## Author contributions

AK performed research, analyzed data and written manuscript. PA assisted to carry out the research work. Soil analysis was done by NP. Nutritional analysis was done by AKJ. Editing of the manuscript was done by VKU, PKM, and RG. Finalization of the manuscript and research were carried out under the guidance of AVS. All authors contributed to the article and approved the submitted version.
