# Supplementary material for: Credibility assessment of cold adaptive Pseudomonas jesenni MP1 and P. palleroniana N26 on growth, rhizosphere dynamics, nutrient status, and yield of the kidney bean cultivated in Indian Central Himalaya
Source: Front Plant Sci. 2023 Jan 30;14:1042053. doi: 10.3389/fpls.2023.1042053 (PMC9926967; doi:10.3389/fpls.2023.1042053)
Supplement: Supplementary file 1 [file Table_1.docx]

**Supplementary table 1: Description of bacterial strains used in current study**

| **SN** | **Name of Strains** | **NCBI accession no.** | **NAIMCC accession no.** | **Isolation site** | **Location** | **Elevation** |
| --- | --- | --- | --- | --- | --- | --- |
| 1 | *Pseudomonas jesenii* MP1 | JX310329 | B-01444 | Munsyari | 30.07˚N, 80.23˚ E | 2200 msl |
| 2 | *Pseudomonas palleroniana* N26 | JN055435 | B-01947 | Nainital | 29.23˚N, 79.30˚E | 2084 msl |
